# Extraction and characterization of pectin from watermelon rind using acetic acid

**DOI:** 10.1016/j.heliyon.2023.e13525

**Published:** 2023-02-08

**Authors:** Dawit Mamiru, Girma Gonfa

**Affiliations:** a Department of Chemical Engineering, Addis Ababa Science and Technology University, 16417 Addis Ababa, Ethiopia; b Biotechnology and Bioprocess Center of Excellence, Addis Ababa Science and Technology University, 16417 Addis Ababa, Ethiopia

**Keywords:** Watermelon rind, Pectin, Extraction, Acetic acid, Response surface methodology

## Abstract

In this work, watermelon rind was used for extraction of pectin with acetic acid solution. The effects of pH, temperature and extraction time on the pectin yield were investigated. Response surface based on Box-Behnken model was employed to optimize the extraction parameters. The model shows an optimum pectin yield of 18.21%, which is in agreement with the value confirmed through experiment (18.20%). The moisture content, ash content, degree of esterification, degree of methylation, equivalent weight, methoxy content, and anhydrouronic acid of the extracted pectin were determined. The values of the moisture content, ash content, degree of esterification, degree of methylation, equivalent weight, methoxy content, anhydrouronic acid are 8.42%, 5.1%, 57.30%, 23.5%, 983.9 mg/mol, 7.3% and 72.36%, respectively. The results show watermelon peel can be an alternative source for pectin production with reasonable pectin yield and pectin quality.

## 1 Introduction

Watermelon (*Citrullus lanatus*) is one of the economically important global fruits with a global production of 101 million tonnes in 2020 [1]. Currently, it grows on all continents and in 122 countries [2]. The watermelon constitutes approximately 2% seeds, 30% rind and flesh [3]. The watermelon rind is one of the major solid wastes that is discarded into the environment [3]. More than 90% of the watermelon rind wastes are dumped as a residue into the environment thereby constituting environmental challenges [3]. About 36 million tonnes of watermelon rind were discarded into environment in 2013 [4]. Although there are various reports on the potential application of the watermelon rind for various uses, currently, the waste is not used for any industrial applications [3]. Utilization of this waste is very important for economic value as well as environmental problems. Watermelon contains carbohydrates, proteins, fats, minerals, vitamins, etc. [4]. Carbohydrate is the main constituent of the watermelon rind; hence, it can be used for the recovery of valuable compounds such as pectin. Pectin mainly consists of three polysaccharide motifs: homogalacturonan (HG), rhamnogalacturonan I (RG-I) and rhamnogalacturonan II (RG-II) along with neutral sugars such as rhamnose, arabinose, galactose and xylose along with long chain galacturonan segments [5].

Pectin is used in food products, cosmetics, pharmaceuticals, medicines and textiles. It serves as gelling/thickening agent and stabilizer in food industries due to its ability to form aqueous gels. It is used in jams, jellies, fruit drink concentrates, fruit juice, desserts and fermented dairy products [6]. Pectin is also effective in stabilizing ices, sherbets and ice cream [7]. In fruit juice products, pectin can act as a thickener and stabilizer to prevent the separation of the pulp [8]. On the other hand, addition of pectin to tomato juice or ketchup makes make them viscous and good appearance. It also assists in stabilizing and emulsifying salad dressing [8], preventing formation of heavy curd milk and preserving the texture in cheese during heating. In bakery products, pectin improves the texture, yield and moisture-holding capacity of dough. Pectin can be used for preparation of biopolymer that can be used for various applications such as edible film packages [9].

Commercial pectin is extracted from citrus (lemon, lime, orange) peels, apple pomace and sugar beet pulp. Currently, 85.5% of the pectin in the global market is produced from citrus peels, 14.0% from apple pomace and the remaining 0.5% from sugar beet pulp. From the citrus peels, the contributions of lemon, lime and orange peels are 56%, 30% and 13%, respectively [10]. Although these residues have served as the main source of commercial pectin, an increase in global pectin demand, susceptibility of citrus plants to diseases and newly emerging applications of pectin are driving the need for searching alternative sources. The global demand for pectin has been increasing and is expected to increase in the future. The global pectin market was USD 656 million in 2010, and the value increased to USD 958 million in 2015 [11]. The global pectin market is expected to increase by 7.3% during 2018–2023 [11]. In addition to increasing pectin global demand, citrus production is frequently affected by the citrus diseases such as canker, greasy spot, and black spot which affect pectin supplies [12]. Recently pectin has been used for biopolymer, biomedical and drug delivery applications [13]. It has been also used as adsorbent [14,15] and as a green corrosion inhibitor [16]. Therefore, the search for additional pectin sources is important and timely.

Biomass residues such as banana peel, coffee pulp, soy hull, papaya peel, sunflower head, mango peel, watermelon rind, etc. have attracted attention from both academia and industry. In the present work, watermelon rind was used for extraction of pectin using acetic acid solution. Wet watermelon rinds contain 19–21% pectin [17]. Moreover, pectin extracted from watermelon rinds shows a high degree of methyl esterification (about 60%) [4]. Further, it shows higher viscosity and good foaming and emulsifying properties which are important characteristics of emulsifier and stabilizing agents [4]. Previously, pectin extraction from watermelon was investigated with hydrochloric acid and citric acid [18]. In this work, acetic acid was used for extraction of pectin from watermelon rind using acetic acid solution and its physiochemical, morphological and other related properties were studied. The extraction parameters (temperature, pH and extraction time) were optimized using response surface methodology (RSM). RSM is an important tool for optimization extraction parameters to obtain optimum pectin yield [19]. Extraction conditions and nature of acid used for extraction significantly affect the characteristics and yield of pectin. Proximate analyses for the raw watermelon rind were also carried out since its one of the important factors which affect the final product.

## 2 Materials and methods

### 2.1 Materials

All chemicals used in this work were analytical grade. Acetic acid, ethanol, sodium hydroxide, sodium chloride, hydrochloric acid and phenolphthalein were used for extraction and characterization of pectin. The red watermelon was supplied by Debre Zeit Agricultural Research Center, Bishoftu, Ethiopia. The watermelon rinds were separated from the fruit, cut into pieces, washed and dried in a vacuum oven at 45 ^°^C for 48 h. The dried watermelon peel was ground in mesh size of 100 μm and packed in polyethylene bag at ambient temperature in addition to that stored for extraction process.

### 2.2 Pectin extraction

Pectin extractions were carried out using 1 M aqueous acetic acid with solid to liquid ratio (1: 30), at pH (1.0–5.0), extraction temperature (60–120 °C) and extraction time (50–110 min). Watermelon rind powder (15 g) was added to a 500 mL flask and 450 mL 1.0 M aqueous acetic acid was added to the flask. The solution was then heated to the required temperature under constant mixing for a given time. Sodium hydroxide was used to adjust pH of the solution. The solution was separated by applying muslin cloth. Then, ethanol was added to the solution at ethanol to solution ratio of 2:1 (v/v) and the mixture was stirred for 10 min and kept overnight to allow pectin coagulation. The coagulated pectin was separated by muslin cloth and washed three times using ethanol. The separated pectin was dried in a vacuum oven at 45 °C for 24 h and then cooled at room temperature. Finally, the dried pectin was ground into fine particles and stored in a moisture-proof airtight bag until further use.

### 2.3 Characterizations

#### 2.3.1 Watermelon rind proximate analysis

The proximate analyses of watermelon rind were carried out according to Association of Official Analytical Chemistry [20]. Moisture content of watermelon rind was determined by drying the pectin sample in an oven at 105 °C. Ash content was determined by treating the dried pectin sample in a furnace at a temperature of 600 °C for 3 h. Crude protein content of watermelon peel sample was analyzed using Kjeldahl method. Crude fiber content was analyzed using gravimetric method and fat content was determined using Soxhlet method. Carbohydrate content was estimated using Equation (1)

(1) Ch(%)=(100−Ms−Cp−Cf−As)%

where, *Ch*, *Ms*, *Cp*, Cf and *As* are the carbohydrate, moisture, crude protein, crude fat and ash contents, respectively.

#### 2.3.2 Physiochemical properties of pectin

Moisture and ash contents of the watermelon rind pectin were determined using the method developed by Association of Official Analytical Chemistry [20]. The equivalent weight of the extracted pectin was determined by Ranganna method [21]. Here, pectin powder (0.5 g), ethanol (5 mL) and distilled water (100 mL) were added to 500 mL conical flask. Then, sodium chloride (1.0 g) was added to sharpen the point of titration and six drops of phenolphthalein red indicator were added. Finally, the mixture was titrated with 0.1 N sodium hydroxide until the pink colour was obtained. The neutralized solution was kept for the determination of methoxy content. The equivalent weight was determined using equation (2)

(2) $$Equivalent\;weight\;\left(g/mol\right)=\frac{Weight\;of\;sample\left(g\right)}{mL\;of\;alkali\times normality\;of\;alkali}\times 1000$$

To determine the methoxy content, 25 mL (0.25 M) sodium chloride was added to the solution and the solution was stirred and allowed cool at ambient temperature for 30 min. Then, 25 mL (0.25 M) hydrochloric acid was added and titrated again with 0.1 N of sodium hydroxide to the same endpoint as equivalent weight. The percent methoxy was determined using equation (3)

(3) $$Methoxy\;content\;\left(\%\right)=\frac{ml\;of\;alkali\times normality\;of\;alkali\times 31}{weight\;of\;sample}\times 100$$

Degree of esterification (DE) was calculated using equation (4)

(4) $$Methoxy\;Content\;\left(\%\right)=\frac{V_{2}}{{V_{1}+V}_{2}}\times 100$$

where, *V*_*1*_ is the volume of titration from equivalent weight and *V*_*2*_ is volume titration from methoxy determination.

The anhydrouronic acid content (AUA) was determined using equation (5)

(5) $$AUA\;\left(\%\right)=\frac{176\;x\;0.1\;(N)\;x(V_{1}\left(mL\right)+V_{2}(mL)}{1000\;x\;weight\;of\;sample\left(mg\right)}x100$$

where, *V*_*1*_ is sodium hydroxide used in determination of equivalent weight content and *V*_*2*_ is the sodium hydroxide used in the determination of methoxyl content.

### 2.4 FTIR and morphological analyses

Fourier-transform infrared spectroscopy (FTIR), Scanning electron microscope (SEM), X-ray Powder Diffraction (XRD) and Thermogravimetric Analysis (TGA) were used to analyze the raw watermelon rind and extracted pectin and the changes observed during extraction of pectin. The results were also used for comparison of the characteristics of the current pectin with pectin obtained from other sources. The FTIR spectrum was taken using Fourier transform infrared spectroscopy (Thermo Scientific iS50 ABX). The FTIR spectrums of both dried watermelon rind and extracted pectin were taken over 4000 and 400 cm^−1^ wavenumbers. The morphology of the watermelon rind and its pectin were investigated using SEM (FEI, INSPCT-F50, Germany). The crystal structures of the samples were analyzed using XRD (XRD-7000). A differential thermogravimetric analyzer (DTG60H) was used to analyze the thermographic properties of watermelon rind powder and extracted pectin.

### 2.5 Response surface methodology

A 3- factor- 3- level Box-Behnken design (BBD) based response surface methodology (RSM) was employed to investigate the effect of three factors (pH, temperature and extraction time) on the response (pectin yield). A total of 15 experimental runs were conducted with three center points. A second-order polynomial model was used to fit the response variable with the factors (Equation (6)).

(6) $$Y=\beta_{0}+\sum \beta_{i}X_{i}+\sum \beta_{ii}X_{i}^{2}+\sum \beta_{ij}X_{i}X_{j}$$

where, Y is the pectin yield (%), *X*, is the factors and β_i_ is the coefficient. The developed model was evaluated by analyzing the values of regression coefficients, ANOVA, F- and P-values. Finally, three-dimensional response surface plots were drawn to visualize the individual and the interaction between the effects of the independent variables on the response. RSM design was carryout with a Design Expert (Version 12.0, Stat-Ease Inc., USA). The proposed model was validated by performing new tests under optimal condition. The pectin yield was calculated using equation (7)

(7) $$Y\left(\%\right)=\frac{m_{2}}{m_{1}}x100$$

where, *m*_*1*_ and *m*_*2*_ are the weight of dried watermelon rind and dried pectin.

## 3 Results and discussion

### 3.1 Characterization

#### 3.1.1 Watermelon rind proximate analyses

The proximate analysis of the watermelon rind was determined to mainly see the composition of its ash, carbohydrate, protein, fiber and fat contents. These parameters affect the quality and quantity of the extracted pectin. The moisture content of the dried watermelon rind is 6.20%, which is lower than the values reported for other red watermelon rinds (Table 1). The ash (fixed mineral residue) of the studied watermelon rind is 7.88%, which is lower than the value reported by other authors. The lower ash content in the current watermelon rind makes it suitable for its utilization for pectin extraction as it may result in low ash content pectin. The carbohydrate and crude protein contents are comparable with the value reported for other watermelon rinds. Significant variations were observed for fiber contents and fat contents. This variation could be due to the different maturity levels of the watermelon.

**Table 1:** Proximate analysis of dry watermelon rind.

	This work	[22]	[23]	[24]
Moisture content (%)	6.20	9.82	10.61	10.72
Ash content (%)	7.88	17.10	13.09	12.61
Crude protein content (%)	11.53	10.83	11.17	11.21
Crude fiber content (%)	1.00	15.79	17.28	–
Crude fat content (%)	15.00	–	2.44	2.38
Carbohydrate content (%)	58.39	59.59	56.02	73.18

#### 3.1.2 Pectin physiochemical properties

The physicochemical properties of the pectin extracted from the watermelon rind are shown in Table 2. The moisture content of the current pectin is 8.42%, which is in the range recommended by pectin producers. The maximum commercial pectin moisture content recommended by the international pectin producer association is 12% [25]. The moisture content of the current pectin is comparable with the value reported for dragon fruit peel pectin (7.81%) [26]. Lower pectin water content is desirable as it enhances its shelf life. The ash content of the current pectin is 5.10%, which is much lower than the maximum value (10%) recommended by the international pectin producer association [25]. The ash content of the current pectin is lower than the value (19.4%) reported for pectin extracted from *Hylocereus polyrhizus* peels [26]. The lower the ash content of pectin shows its higher purity. The varieties of watermelon, method of pre-treatment and extraction methods affect the ash content of the extracted pectin.

**Table 2:** Physiochemical properties of pectin extracted from watermelon rind pectin.

Characteristics	Value obtained
Moisture content (%)	8.42
Ash content (%)	5.10
Equivalent weight (mg\mol)	983.90
Methoxy content (%)	7.30
Degree of esterification (%)	57.30
Anhydrouronic acid (% AUA)	72.36

The equivalent weight shows the total content of free galacturonic acid in pectin. The equivalent weight of the extracted pectin is about 984 mg/mol which is higher than the minimum value (400 mg/mol) recommended by the international pectin producer association [25]. The equivalent weight of the current pectin is higher than the value reported (356 g/mol) for pectin extracted from watermelon rind using sulfuric acid solution [27], and the value obtained (578 g/mol) for pectin extracted from banana peels with citric acid [28]. It is in agreement with the range of equivalents reported for pectin extracted from apple pomace (833–1666 g/mol) [29] and pectin extracted from banana peel (43–1456 g/mol) [28]. The equivalent weight mainly depends on pectin extraction process (pH, acid used, etc.) and the number of free acids available on pectin which varies with the nature of the pectin sources. Higher equivalent weight indicates higher gel-forming properties of pectin.

Methoxy content shows the degree of methylation and it determines the capacity of pectin to form gel and may suggest strong cohesive and adhesive characteristics of the pectin [4]. Pectin is categorized into two types depending on its methoxy content. High methoxy pectin (HMP) contains higher methoxy (>7.12%) and low methoxy pectin (LMP) contains methoxy (2.5–7.12%) [25]. The methoxy value of the current pectin is 7.30%, hence, it is grouped under high methoxy pectin. The methoxy value of the current pectin is higher than the values reported (3.86–5.97%) for pectin extracted from banana [28] and apple pomace (2.23–6.21%) [29]. Similarly, pectin can be classified into two based degrees of esterification (DE): a high degree of esterification (DE) and a low degree of esterification. DE is the ratio of esterified galacturonic acid groups to total galacturonic acid groups present in the pectin and it is an important property that determines the gelling nature of pectin. Pectin with less than 50% DE is classified as low methoxyl pectin (LMP) while the one with greater than 50% esterification is called high methoxyl pectin (HMP) [29]. The DE of the current pectin is 57.30%, which is categorized under a high degree of esterification. The DE of this pectin is less than the values reported for pectin extracted from banana (63.15–72.03%) [28] and dragon fruit (63.74%). On the other hand, The DE of the current pectin is higher than the values reported for pectin extracted from apple pomace (22.15–52.51%) [29]. The anhydrouronic acid content (AUA) of the current pectin is 72.36% which is comparable to the values reported for pectin extracted from various sources. Higher AUA content indicates the purity of the extracted pectin and AUA value of 65% is recommended for pectin used as food additives and for pharmaceutical purposes [28]. In general, methoxy content, degree of esterification and anhydrouronic acid content not only depends on the nature of the pectin source but also depends on pectin extraction conditions (type of acid used, pH of the solution, extraction time, temperature, etc.) [30].

#### 3.1.3 Fourier-transform infrared spectroscopy

**Fig. 1:**
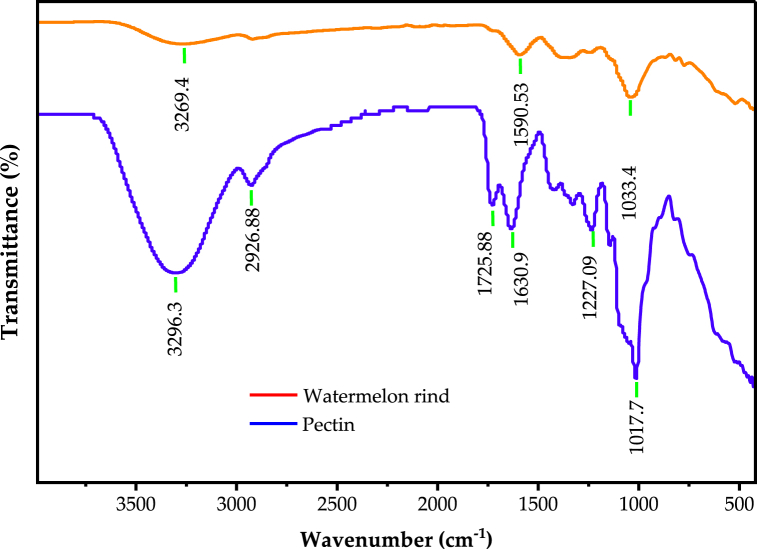
FTIR spectrum of dried watermelon rind and extracted pectin.

The Fourier-transform infrared spectroscopy (FTIR) spectrum of the dried watermelon rind and the extracted pectin is shown in Fig. 1. The FTIR spectrum was taken for sample extracted with acetic acid at pH (2.0), temperature (90 °C) and time (100 min). For dried watermelon powder, band at 3269.4 cm^−1^ represents the OH stretch of water molecules in the watermelon rind. The very weak around 2900 cm^−1^ corresponds to C–H stretching vibrations. These peaks became more pronounced in the FTIR of the pectin. The peak observed between 3990 and 2990 cm^−1^ corresponds to O–H stretching absorption hydrogen bonding of the galacturonic acid (GalA) backbone [31]. The band around 2926 cm^−1^ is the characteristic band for the CH stretching vibration from –CH, –CH_2_, and –CH_3_, methyl esters of galacturonic acid in polysaccharide components [32]. The peaks at 1726 and 1631 cm^−1^ correspond to C=O of esterified carboxylic groups (-COOCH_3_) and free carboxylic groups (-COOH), respectively [31]. The spectra observed between 1300 and 900 cm^−1^ correspond to the ether R-O-R and cyclic C–C ring linkages of the pectin structure [32]. The peaks observed in the extracted pectin are more pronounced compared to dried watermelon rind. This is because on extraction the pectin is concentrated by improving other watermelon rind components and the FTIR functional group becomes increased.

#### 3.1.4 X-ray diffraction

**Fig. 2:**
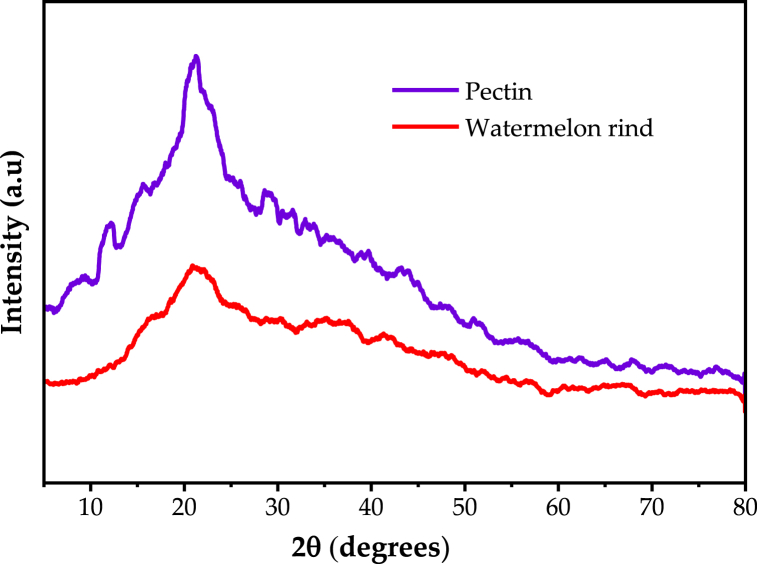
X-ray diffraction (XRD) of watermelon rind and extracted pectin.

X-ray diffraction (XRD) analyses were performed both for dried watermelon rind and the extracted pectin. X-ray diffraction (XRD) analyses were used to study the pectin structure (amorphous or crystalline) of the rind and the pectin. XRD spectrum was taken for sample extracted with acetic acid at pH (2.0), temperature (90 °C) and time (100 min) was obtained. XRD pattern of the watermelon rind and the extracted pectin are shown in Fig. 2. Watermelon rind and the extracted pectin show a weak characteristic diffraction peak at 2θ of 21.3°. Both the watermelon rind and pectin generally show amorphous nature; however, the crystallinity slightly improved for extracted pectin. Moreover, a small peak was observed at 2θ of 12.1° for pectin whereas no peak was observed for watermelon rind at this point. This shows slight increase crystallinity for pectin compared to watermelon rind. Similar observations were reported by previous reports [29,33].

#### 3.1.5 Thermogravimetric analysis

**Fig. 3:**
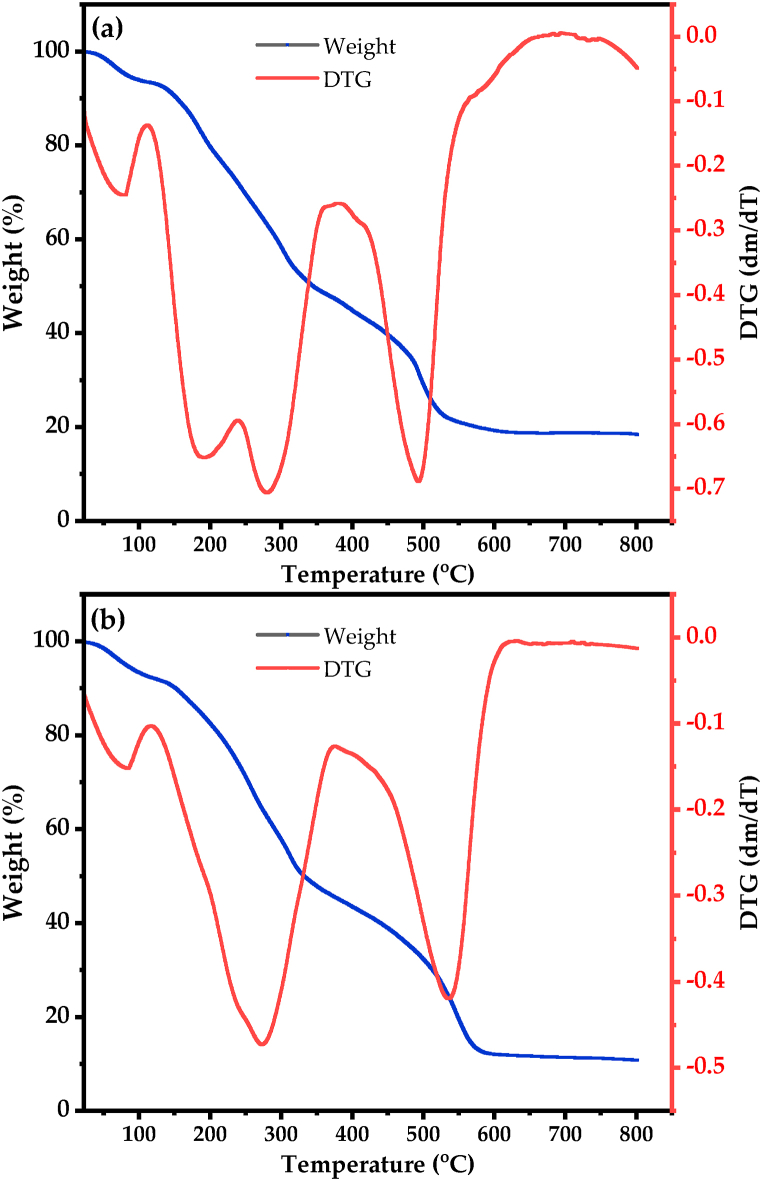
Thermogravimetric analyses of watermelon rind and extracted pectin. (a) dried watermelon rind and (b) extracted pectin.

The thermal stabilities of dried watermelon rind and extracted pectin were investigated using thermogravimetric analysis. The thermogravimetric was performed for sample extracted with acetic acid at pH (2.0), temperature (90 °C) and time (100 min). Fig. 3 shows the TG-DTG curves of the dried watermelon rind and extracted pectin. Fig. 3(a) shows the TG-DTG curve of the dried watermelon rind. Fig. 3(b) shows the TG-DTG curve of the extracted pectin. The thermogravimetric analysis (TGA) results suggest that the weight loss of both the watermelon rind and the extracted pectin can be divided into three regions. In the first region (up to 140 °C) mostly water and other volatile are removed from dried watermelon rind and the extracted pectin [34]. In the second region (150–330 °C), decomposition of the main chain of the pectin and organic compounds in watermelon rind will occur. In the third step (330–800 °C), decomposition of secondary decarboxylation with acid side group and carbon in the ring molecules occurred. Both the dried pectin rind and the extracted pectin show similar thermal decomposition characteristics; however, the degree of decomposition slightly varies. The remaining mass of watermelon rind after decomposing at 800 ^°^C was 18.5%, while the value for extracted pectin is about 10.5%.

#### 3.1.6 Scanning electron microscope

**Fig. 4:**
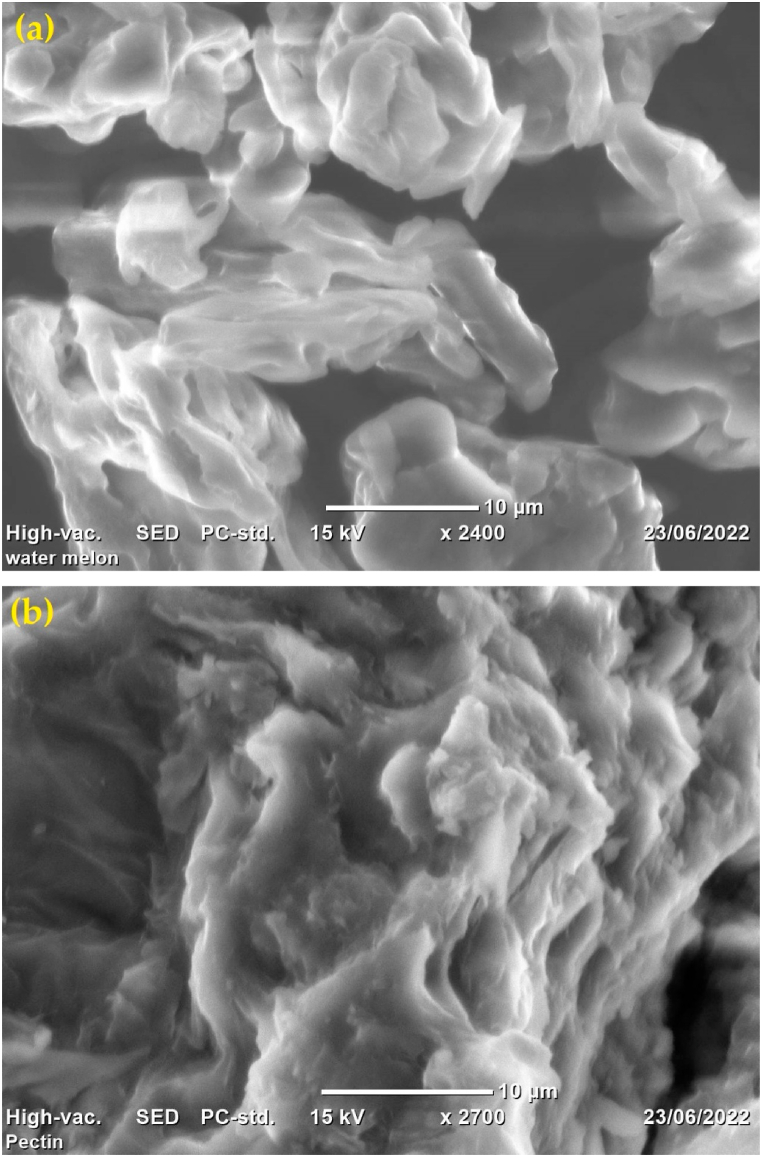
SEM images of dried watermelon rind and extracted section. (a) watermelon rind, (b) pectin.

Scanning electron microscope (SEM) images of dried watermelon rind and extracted pectin are depicted in Fig. 4. Fig. 4(a) shows the SEM image of watermelon rind while Fig. 4(b) shows the SEM image of the extracted pectin. The SEM image was taken for the sample extracted with acetic acid at pH (2.0), temperature (90 °C) and time (100 min). The SEM of watermelon rind contains larger smooth granules with larger void spaces. On the other hand, pectin consists of irregular and filamentous structures. The surface morphology of the extracted pectin may vary extraction conditions.

### 3.2 Pectin yield

**Fig. 5:**
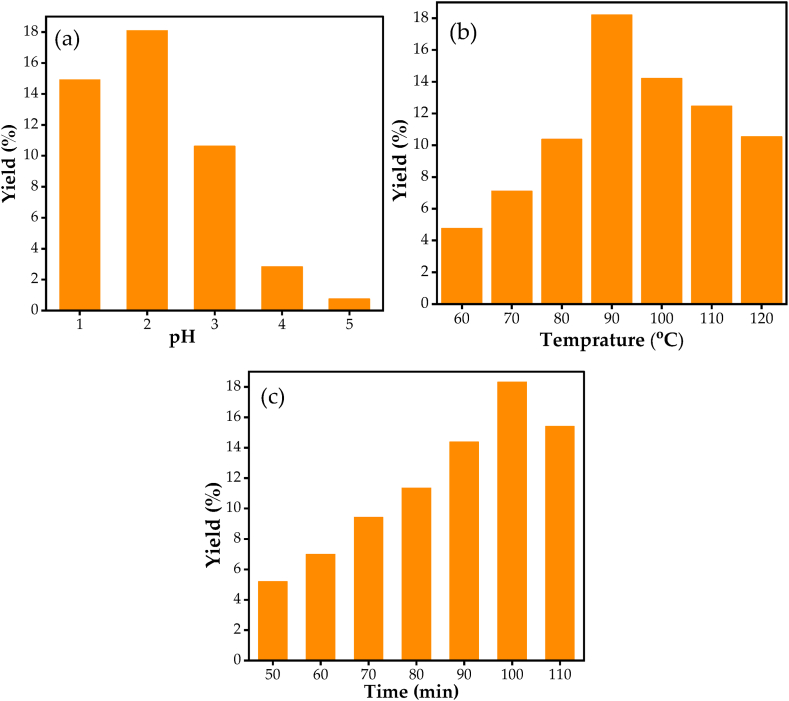
Effect extraction parameters on pectin yield. (a) effect of pH at 100 °C and 100 min extraction time, (b) effect of temperature at pH of 2.0 and 100-min extraction time and (c) effect of extraction time at pH of 2.0 and 100 °C.

Pectin yield depends on various factors, such as pH, temperature, extraction time, types of acid used, and the nature of pectin sources. In this work, the effect of pH, temperature and extraction time were investigated (Fig. 5). The effect of pH on pectin yield is shown in Fig. 5(a). The yield of pectin increases as pH increases from 1.0 to 2.0, however, a further increase in pH reduces the yield. A maximum yield of 18.1% yield was observed at a pH of 2.0 and the value was reduced to 0.74% at pH of 5.0. This shows pH is an important factor in the extraction of pectin. A similar observation was reported by Lee and Choo [18], where higher pH significantly reduced the yield as well as the quality of the extracted pectin. Low pH may promote quick disruption of hydrogen bonds and ester linkages between pectin and cell wall which increase the rate of diffusion of pectin and pectin extraction. The effect of extraction temperature on pectin yield is depicted in Fig. 5(b). The yield increases as temperature increases and attains a maximum yield of 18.21% at 90 °C. However, a further increase in temperature reduced the yield to 10.53% at 120 °C. Higher temperature may cause decomposition of pectin and result in lower pectin yield. The effect of extraction time on pectin yield is indicated in Fig. 5(c). The pectin yield increases as extraction time reach 100 min and a further increase in time resulted in a reduction of the yield. Longer exposure of extracted pectin to acid media affects the extracted pectin and results in the reduction of the yield [35].

### 3.3 Response surface methodology

In this work, response surface methodology based on Box Behnken design (BBD) was employed to determine the optimum condition of the three factors (pH, temperature and extraction time). A total of 15 experiments were carried out to generate the response surface. A second-order model was fitted to the experimental data to develop a numerical relationship between the three factors and the response (equation (8)). The analysis of variance (ANOVA) for the response surface quadratic model is indicated in Table 3. The p-values less than 0.05 are represented as significant terms. The p-values for most of the independent and interaction factors are less than 0.0001 and R^2^ value is 0.9955. This implies the model sufficiently predicts the pectin extraction yield from the three extraction parameters. The second-order polynomial model for pectin extraction yield is shown in equation (8). The experimental results and the values that are predicted by the model are presented in Table 4. These predicted and excremental values at all combinations are not far from each other which indicates the model can satisfactorily predict the response.

(8) $$Y=18.3-0.54A+0.2262B+0.2263C+0.3025AB-0.1275AC-0.3150BC-1.96A^{2}-1.31B^{2}-1.55C^{2}$$

where; *Y* is pectin yield, *A* is the pH, *B* is temperature and *C* is the extraction time. Based on the developed model the optimum pectin yield of 18.21% was observed at pH (2.3), temperature (93 °C) and extraction time (101 min). The experimental pectin yield value at this condition was 18.20%. Three measurements were made and average value was reported for the experimental pectin yield at the optimum extraction condition. This shows the model can predict the optimum pectin yield under the current extraction conditions.

**Table 3:** ANOVA analysis of response surface quadratic model.

Source	Sum of Squares	df	Mean Square	F-value	p-value	
Model	29.59	9	3.29	342.68	<0.0001	significant
A-pH	2.33	1	2.33	243.13	<0.0001	
B-Temperature	0.4095	1	0.4095	42.68	0.0013	
C-Time	0.4095	1	0.4095	42.68	0.0013	
AB	0.3660	1	0.3660	38.15	0.0016	
AC	0.0650	1	0.0650	6.78	0.0481	
BC	0.3969	1	0.3969	41.37	0.0013	
A^2^	14.15	1	14.15	1474.54	<0.0001	
B^2^	6.34	1	6.34	660.38	<0.0001	
C^2^	8.87	1	8.87	924.52	<0.0001	
Residual	0.0480	5	0.0096			
Lack of Fit	0.0280	3	0.0093	0.9325	0.5547	Not significant
Pure Error	0.0200	2	0.0100			
Cor Total	29.64	14				
R^2^	0.9984					
Predicted R^2^	0.9834					
Adjusted R^2^	0.9955

**Table 4:** Design matrix for the three independent variables for extraction of pectin from watermelon rind with experimental and predicted yield values.

Runs	Experimental Parameters			Yield (%)	
	pH	Temperature (^°^C)	Time (min)	Actual	Predicted
1	3	80	100	13.98	13.97
2	2	100	110	15.65	15.58
3	1	90	110	15.75	15.78
4	2	80	110	15.74	15.75
5	3	90	90	14.21	14.16
6	2	90	100	18.20	18.30
7	3	90	110	14.35	14.35
8	3	100	100	14.95	14.97
9	1	100	100	15.72	15.65
10	2	100	90	15.77	15.76
11	2	90	100	18.24	18.30
12	2	90	100	18.30	18.30
13	2	80	90	14.60	14.67
14	1	90	90	14.98	14.96
15	1	80	100	15.72	15.65

The pectin extraction yield from watermelon yield using acetic acid under the current extraction process conditions is compared with other pectin sources. The current pectin yield is higher than the values reported for papaya powder (2.6%) [36], banana peels (2.18%) [37], pumpkin (11.35%) [38], passion fruit peel (12.67%) [39], green tea leaf (8.5%) [40], carrot pomace (15.2%) [41] and musa balbisiana (8.99%) [42]. It is comparable with the values reported for grapefruit peel (17.92%) [43], jackfruit rind (17.63%), pistachio green hull 18.13% [44] and apple pomace 15.75% [45]. The current pectin extraction yield was also compared with previous works on the extraction of pectin from watermelon rinds. The current pectin yield is higher than the value reported by various authors. The highest pectin yields reported were 17.9% for citric acid [46], 13.4% for hydrochloric acid [47] and 11.25% for sulfuric acid [48].

**Fig. 6:**
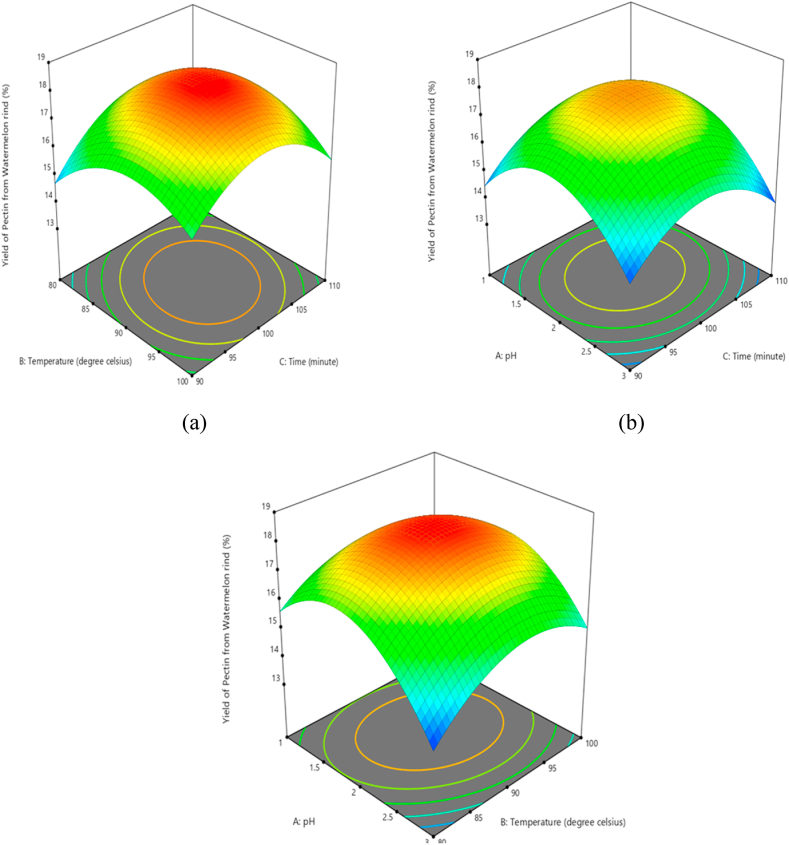
3D response surface plot for interaction effects between the factors. (a) temperature and time, (b) pH and extraction time, (c) pH and temperature.

Fig. 6 shows the three-dimensional response surface representations of the pectin extraction yield at various process conditions. Each contour graph reveals the effect of two parameters on the yield of pectin extracted while holding the third parameter at a constant level. The 3D surface plots were drawn between any two extraction variables by keeping the other variable constant. Fig. 6(a) shows the effects of extraction time and extraction temperature at constant pH of 2.0. It can be observed that an increase in time and temperature leads to a gradual increment of pectin percentage yield. However, further increments in temperature and time reduced the pectin yield. At higher temperature and extraction time pectin may be affected by the acid resulting in lower yields. Fig. 6(b) shows the effect of pH and extraction time on pectin yield. Similarly, the pectin yield initially increases with pH and extraction time, however, further increases in pH and time results introduction of the yield. Lower pH raises the H^+^ concentration in the medium and stimulates the hydrolysis of protopectin and suppress highly hydrated carboxylate groups leading to higher pectin yield [30]. The effect of pH and activation temperature is indicated in Fig. 6(c). Similarly, pectin yield initially increases with increasing extraction time and temperature, however, further increase in the parameters results in the reduction of yield.

## 4 Conclusion

In this work, pectin was successfully extracted from dry watermelon rind with acetic acid. Both the watermelon rind and pectin were characterized for morphological and chemical characteristics. The effects of pH of solution, extraction temperature and extraction time on the yield of extracted pectin were found to be significant. The highest extraction yield of 18.20% was observed pH of 2.0, temperature of 90 °C and extraction time of 100 min. Response surface method based on Box-Behnken experimental design was employed to establish the numerical relation between the three pectin extraction parameters (pH, temperature and extraction time and the response (pectin yield) with reasonable accuracy. The model provided 18.21% pectin yield which is comparable is the experimental result. The pectin extracted can be considered as high methoxy pectin (DE 57.30% and Meo 7.30%) and has high quality (AUA >65%). This shows extraction of pectin with acetic acid from watermelon rind can provide reasonable pectin yield with good quality pectin.

## Author contribution statement

Dawit Mamiru: Performed the experiments.

Girma Gonfa: Conceived and designed the experiments; Performed the experiments; Analyzed and interpreted the data; Contributed reagents, materials, analysis tools or data; Wrote the paper.

## Funding statement

This work was supported by the Addis Ababa Science and Technology internal research grant (IG 03/2022).

## Data availability statement

Data will be made available on request.

## Declaration of interest’s statement

The authors declare no conflict of interest.
